# Carbon Dots Derived from Non-Biomass Waste: Methods, Applications, and Future Perspectives

**DOI:** 10.3390/molecules29112441

**Published:** 2024-05-22

**Authors:** Wenjing Chen, Hong Yin, Ivan Cole, Shadi Houshyar, Lijing Wang

**Affiliations:** 1 School of Fashion and Textiles, RMIT University, Brunswick, VIC 3056, Australia; s3981322@student.rmit.edu.au (W.C.); lijing.wang@rmit.edu.au (L.W.); 2 School of Engineering, STEM College, RMIT University, Melbourne, VIC 3000, Australia; ivan.cole@rmit.edu.au (I.C.); shadi.houshyar@rmit.edu.au (S.H.)

**Keywords:** carbon dots, non-biomass waste, environment management, textiles, sustainability

## Abstract

Carbon dots (CDs) are luminescent carbon nanoparticles with significant potential in analytical sensing, biomedicine, and energy regeneration due to their remarkable optical, physical, biological, and catalytic properties. In light of the enduring ecological impact of non-biomass waste that persists in the environment, efforts have been made toward converting non-biomass waste, such as ash, waste plastics, textiles, and papers into CDs. This review introduces non-biomass waste carbon sources and classifies them in accordance with the 2022 Australian National Waste Report. The synthesis approaches, including pre-treatment methods, and the properties of the CDs derived from non-biomass waste are comprehensively discussed. Subsequently, we summarize the diverse applications of CDs from non-biomass waste in sensing, information encryption, LEDs, solar cells, and plant growth promotion. In the final section, we delve into the future challenges and perspectives of CDs derived from non-biomass waste, shedding light on the exciting possibilities in this emerging area of research.

## 1. Introduction

**Figure 1:**
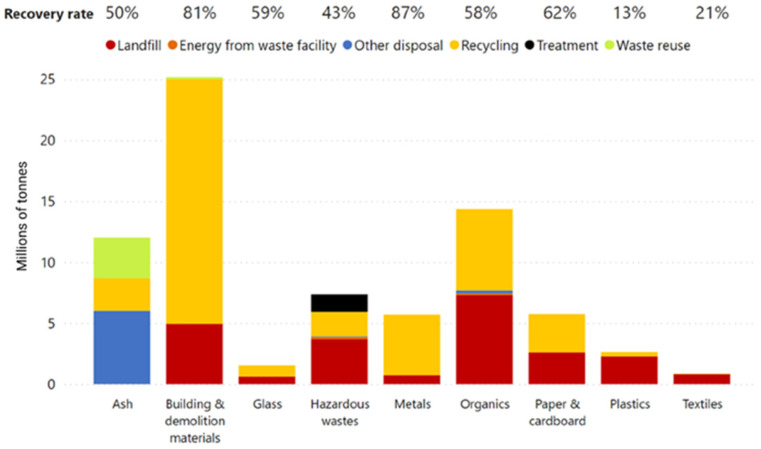
Waste generation and management methods categorized by material type, Australia 2020–2021 [11].

Escalating global greenhouse gas emissions pose a threat to the sustainability of our planet [1]. Waste management practices, including collection and landfill operations, account for approximately 5% of atmospheric greenhouse gas emissions [2,3]. The growing volume of waste generation presents a significant challenge in terms of responsible disposal and environmental protection. In this context, various strategies, including reducing, reusing, recycling, and recovery, have been implemented to promote resource efficiency and environmental friendliness and develop a competitive low-carbon economy [4,5]. However, a considerable portion of waste still ends up in landfills. Non-biomass wastes, such as plastics and textiles, when consigned to landfills, can cause long-term ecological impacts on the planet, as they could persist in the environment for centuries or even millennia [6,7,8,9]. The issue is further exacerbated by the bans on the export of waste materials like plastic, paper, glass, and tyres to countries where value may be added to the waste materials. Figure 1 shows waste generation and management methods categorized by material types. Notably, the highest recovery rate is for metals at 87%, closely followed by building and demolition materials at 81%. In contrast, plastics and textiles exhibit the lowest recovery rates, standing at 13% and 21%, respectively, underscoring the critical need for more effective approaches to address these persistent waste management challenges [10,11].

Carbon dots (CDs) represent a relatively recent entrant in the domain of “zero-dimensional” carbon nanoparticles, characterized by their small sizes from 1 to 10 nm. Due to their excellent optical, physical, biological, and catalytic properties, CDs have been linked to widespread interest and have found diverse applications in fields such as environmental treatment and protection, sensing, drug delivery, bioimaging, fluorescent inks, catalysis, heavy metal ion detection, and more [12,13,14,15,16]. CDs can be synthesized from various precursors, including waste materials. A literature search on the synthesis, properties, and applications of waste-derived CDs was conducted using keywords including waste, rubbish, trash, carbon dots, CDs, carbon quantum dots, and carbon nanodots. As shown in Table 1, there has been a predominant focus in the literature on CDs derived from natural waste, especially biomass residues [14,17,18,19,20,21,22,23,24,25,26,27,28,29,30]. Limited attention has been granted to CDs derived from non-biomass waste materials, such as plastics, sludge, wastepaper, waste kitchen chimney oil, and waste soot (e.g., kerosene fuel soot and candle soot) [14,18,20]. Considering the increasing generation of non-biomass wastes and their non-biodegradable nature, there is a need for a comprehensive review that addresses the conversion of non-biomass waste into valuable CDs. In this review, we first classify non-biomass waste sources according to the 2022 Australian *National Waste Report* (https://www.dcceew.gov.au/sites/default/files/documents/national-waste-report-2022.pdf (accessed on 23 November 2023)) and subsequently summarize the synthesis methods, characterizations, and properties of the CDs prepared from these non-biomass waste materials. This review further provides a survey of the applications of non-biomass-waste-derived CDs, such as sensing, information encryption, LEDs, solar cells, and plant growth promotion. Finally, we outline the challenges ahead and suggest avenues for future work to foster the advancement and commercialization of CDs derived from non-biomass waste.

## 2. Waste Precursors

Non-biomass waste materials include a broad spectrum, such as ash, building and demolition materials, glass, metals, organics, paper and carboard, plastics, textiles, leather, rubber, and various composite materials. Developing innovative methodologies for converting non-biomass waste to CDs has attracted increasing attention from both industry and academy [11].

**Figure 2:**
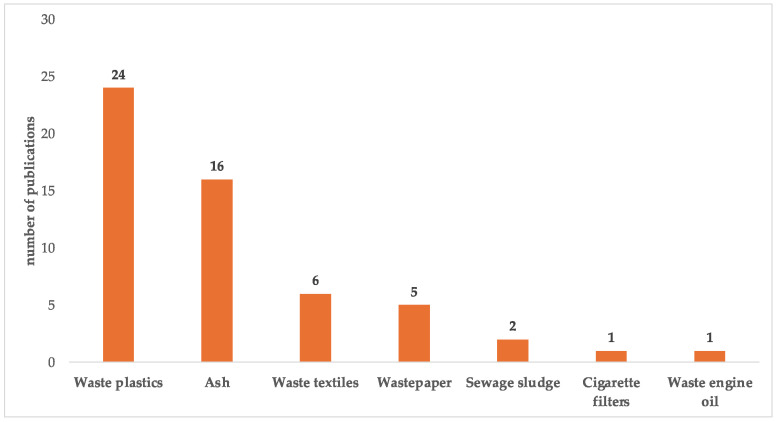
Published papers (up to the end of 2023) on the CDs derived from non-biomass waste based on their precursor materials.

In the realm of CDs, particle size and quantum yield (QY) are important factors that dictate their properties. Generally, a high fluorescent emission efficiency is associated with small particles and a narrow size distribution [32]. Fluorescence QY is a quantitative indicator of the substance’s ability to emit fluorescence, defined as the ratio of emitted photons to absorbed photons [33]. The prevailing opinions attribute the emission of CDs to their surface state, carbon core state, molecule state, and their synergistic effect [34,35,36,37]. Unlike semiconductor quantum dots, the emission wavelength of CDs cannot be tuned by controlling their particle size alone. In contrast, various factors, such as heteroatom doping, solvatochromic effect, concentration-dependent effect, and surface functionalization, all contribute to the optical properties of CDs [38]. A wide range of waste precursors and different synthetic procedures (e.g., hydrothermal, microwave, refluxing, and pyrolysis) result in a wide size distribution in the CD product. Due to the unknown composition of the waste precursors, the prepared CDs are also accompanied by unreacted impurities or by-products that interfere with their pristine emission properties. Therefore, conventional CDs derived from waste normally show a wide emission wavelength with a full width at half-maximum of more than 100 nm [39,40,41,42]. In this literature review, a systematic search method was employed to gather existing research on CDs derived from non-biomass waste, focusing on their precursor materials (including ash [32,39,42,43,44,45,46,47,48,49,50,51,52,53,54,55], waste plastics [40,56,57,58,59,60,61,62,63,64,65,66,67,68,69,70,71,72,73,74,75,76,77,78], wastepaper [79,80,81,82,83], waste textiles [84,85,86,87,88,89], cigarette filters [90], sewage sludge [41,91], and engine oil) [92]. As shown in Figure 2, 55 relevant articles met our rigorous inclusion criteria. There are 24 and 16 articles on waste plastic- and ash-derived CDs, accounting for 44% and 29% of the total publications, respectively. Additionally, the numbers of research articles on CDs from waste textiles and wastepaper are six and five, respectively.

### 2.1. Ash

Ash is a residual by-product of coal-fired power generation [11], and ash waste has surged due to population growth and economic development. Traditional methods of managing coal ash waste are dry storage and wet disposal. Recently, resource recovery has offered a more sustainable alternative with significant environmental, social, and economic benefits, thus attracting considerable attention [93]. The composition of ash waste depends on the source material being burned, such as coal and plants, while the main chemical constituent in ash is carbon [94]. Various types of CDs have been extensively reported, including those derived from candle soot, cigarettes, oil fly ash, diesel soot, and toner powder, from 2011 to 2023 [32,39,42,43,44,45,46,47,48,49,50,51,52,53,54,55]. Table 2 lists the comprehensive details of the CDs derived from ash waste, including the methods utilized, particle sizes, and QY.

The ball milling technique is a typical top-down method to fabricate nanomaterials and has been widely used to generate carbon nanoparticles (CNPs) from ash waste. CNPs produced via ball milling generally exhibit large sizes. CNPs with cluster sizes ranging from 22.3 nm to 35 nm were successfully synthesized from oil fly ash by ball milling in a dry medium, followed by sonication in liquid media containing deionized water and nitric acid. In another instance, CNPs with sizes less than 100 nm were synthesized from oil fly ash, using high-energy ball milling in an acetic acid medium [43,44]. In contrast, certain direct burning methods have produced significantly smaller CDs. For example, CDs within a size range of 4.5 to 7.0 nm were derived from cigarette smoke, while even smaller-sized CDs with narrower size distribution (2.0–4.0 nm) were obtained from burning of flammable organic materials, such as ethanol, n-butanol, domestic candle, and benzene [32,50].

Chemical oxidation is another method to prepare CDs from ash. CDs with a size of 2–3 nm and a high QY of 3–5% were synthesized using fullerene carbon soot through a nitric acid refluxing approach [54]. Coals from the northeastern coalfield (Cenozoic age) in India, including Coal-NK, Coal-NG, Coal-T60, and Coal-T20, were used in a wet-chemical ultrasonic stimulation process to produce CDs with different sizes (1–4 nm, 1–6 nm, 2–5 nm, and 10–30 nm) and QY values ranging from 3 to 14%. This method stands out for its environmental friendliness and cost-effective coal feedstocks [95].

Microwave pyrolysis has emerged as a predominant method for CDs synthesis owing to its high efficiency, energy conservation, and straightforward equipment operation [98,99,100]. In a recent publication, CDs with an average size of 2.1 nm were derived from waste toner powder via microwave pyrolysis in an ethanol solvent. These CDs demonstrated a yellow emission at 557 nm upon excitation at 300 nm, holding promises for potential LED applications [55].

Emissions from vehicles have a detrimental impact on the global environment and accelerate climate change [101,102,103]. To address these issues, numerous policies, legislation, and enforcement strategies were developed to control exhaust emissions from vehicles [104,105]. Recent research by Chaudhary et al. (2022) showed that CDs with just 2 nm in size could be prepared using bike pollutant soot and distilled water through a hydrothermal process [45]. Additionally, Soxhlet-purification was effectively employed to transform harmful diesel soot into larger CDs with an average size of 20–30 nm and a QY of ~8% [39].

### 2.2. Waste Plastics

**Figure 3:**
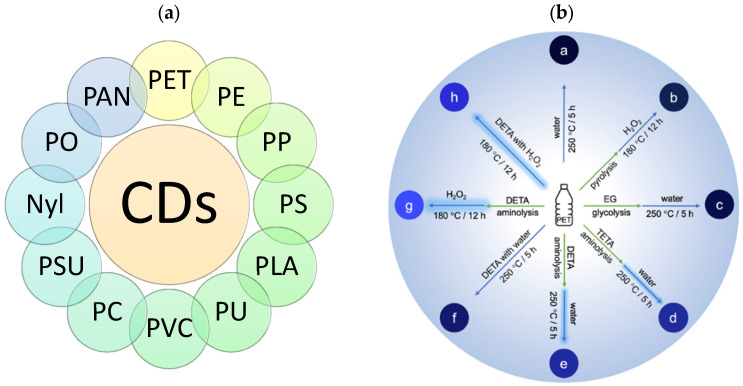
(**a**) Resources to fabricate CDs from plastics during 2018–2023 [40,56,57,58,59,60,61,62,63,64,65,66,67,68,69,70,71,72,73,74,75,76,77,78], Note: PET: polyester, PE: polyethylene, PP: polypropylene, PS: polystyrene, PLA: polylactide, PU: polyurethane, PVC: polyvinylchloride, PC: polycarbonate, PSU: polysulfone, Nyl: nylon, PO: polyolefins, and PAN: polyacrylonitrile. (**b**) CDs from PET via Various Conversion Routes [56]. Reproduced with permission from Elsevier.

Plastics play a critical role in the global economy but create increasing environmental concerns due to non-biodegradability and recycling difficulties [106,107,108]. It is estimated that a staggering 87% of plastic waste ends up in landfills [11]. Since polymers generally contain abundant carbon chains, repurposing plastics into CDs presents an efficient strategy to transform waste into a valuable resource. The plastic resources used for CD synthesis from 2018 to 2023 are summarized in Figure 3a [40,56,57,58,59,60,61,62,63,64,65,66,67,68,69,70,71,72,73,74,75,76,77,78]. Table 3 lists the main approaches for synthesizing CDs from waste plastics, including the hydrothermal and pyrolysis method. The pyrolysis approach is a common chemical method to convert plastic waste into carbon materials at elevated temperatures. In contrast, the hydrothermal approach is the most widely used method for CD synthesis from plastics due to its lower operating temperature, high yield, ability to obtain CDs with a small size, and narrow size distribution [109,110].

Compared with ashes, waste plastics tend to generate CDs with higher QYs. The main composition of ashes is carbon, with multiple elements, including calcium, magnesium, aluminum, and silicon in their oxide forms [111]. These materials have been subjected to a high temperature calcination and remained stable in these conditions. In contrast, the carbonaceous backbones in plastic wastes are more susceptible to hydrolysis, ring opening, and crosslinking reactions involved in CD synthesis. As a result, various compounds with different types of polar functional groups are produced, leading to a high QY [37,112]. Kumari et al. (2020) fabricated CDs from single-use plastic waste, including plastic bags, cups, and bottles made up of polyethylene, polypropylene, and PET based polymers, respectively [67]. The waste was first heated at 300 °C for 2 h. Afterwards, the calcinated samples were added into 15 mL of deionized water and subjected to hydrothermal treatment at 200 °C for 5 h. The fluorescence QY values for CDs ranged from 60% to 69%, and the CDs prepared from PET-based waste bottles demonstrated the highest QY [67]. As shown in Table 3, most high QY values were obtained from waste PET bottles. It could be related to its carbonaceous backbone with abundant oxygen-containing functional groups that facilitate hydrolysis, condensation, and later carbonization [113]. Research conducted by Wang et al. (2023) converted PET waste bottles into CDs using a direct hydrothermal ammonolysis approach, resulting in CDs with an average size of 2 nm and an impressive QY of 87.36% [54]. In this process, ammonium hydroxide and pyromellitic acid were used as precursors together with PET. The as-prepared PET-CDs were not only successfully doped with nitrogen in the form of pyrrole N structures but also covered with -NH_2_ and -COOH groups on the surface [54]. All these factors act collectively, contributing to their extremely high QY.

Unlike ash waste, which naturally exists in a powdered form to be used for CD synthesis, plastic wastes generally need additional pre-treatment processes to convert their different morphologies into powders suitable for the subsequent reactions. The pre-treatment methods include grinding, alcoholysis, hydrolytic degradation, aminolysis, pyrolysis, thermal treatment, and more [56,58,59]. Some studies have reported the pyrolysis of plastics at a high temperature of 300–400 °C for about 2 h, either in an oven or a microwave, before a hydrothermal process [60,62,65,67,68,72,76]. Chan et al. (2022) used various pre-treatment routes for PET, including pyrolysis, glycolysis, and aminolysis with or without an oxidizing agent (Figure 3b) [56]. The fluorescence properties of CDs derived from waste PET showed that direct hydrothermal synthesis of PET or a combination of pyrolysis or glycolysis pre-treatment resulted in non-fluorescent or weak fluorescent products. Whereas the aminolysis of PET bottle plastics followed by hydrothermal synthesis led to a dramatic increase in fluorescence (ten to one hundred times higher than the original). Adding a small amount of oxidant (H_2_O_2_) to the hydrothermal mixture achieved a conversion yield of 25.3% and a QY of 9.1%.

### 2.3. Waste Textiles and Wastepaper

**Figure 4:**
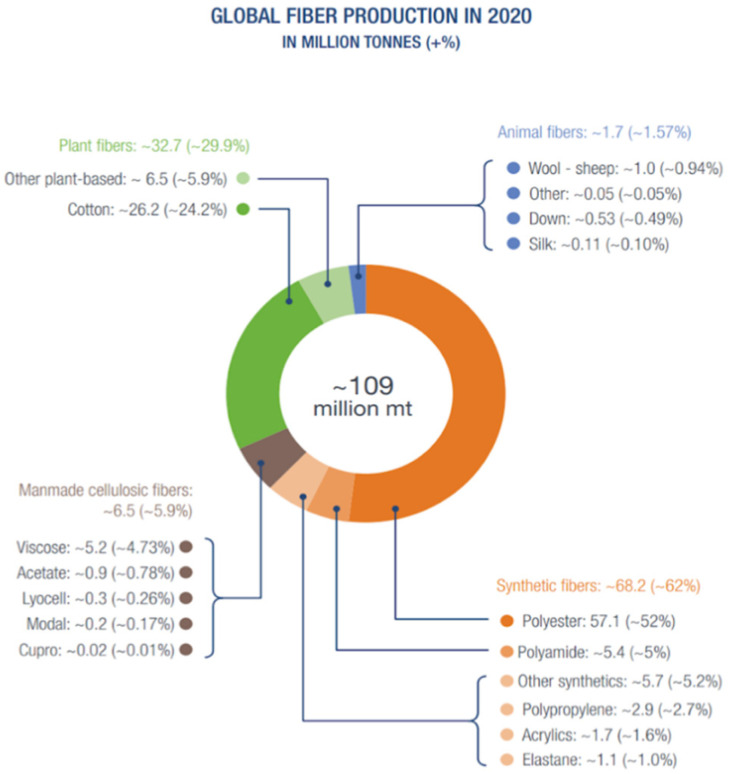
Global fiber productions in 2020 [114].

As shown in Figure 4, the primary sources of fibers in global textile production include synthetic fibers (polyester, polyamide, and PP), manmade cellulose fibers (like viscose and acetate), plant-based fibers (including cotton), and animal fibers (such as wool and silk) [114]. Wastepaper comes from newsprint, magazine, printing and writing papers, and packaging papers. Cellulose is the main chemical component of cotton and paper for synthesizing CDs. Table 4 summarizes the methods, sizes, and QY of CDs prepared from waste textiles, wastepaper, and cellulose.

Among synthetic fibers, PET is a prominent waste material and has been widely used for CD synthesis. Using a pre-treatment step combined with a hydrothermal process, CDs with smaller size and higher QY were obtained from waste PET. CDs derived from terylene waste were successfully generated using a hydrothermal method (at 260 °C for 18 h), resulting in particles with sizes of 2.5–7.0 nm and a QY of 49.36% [87]. Wang et al. (2022) synthesized CDs with sizes ranging from 1.6 to 4.6 nm and a high QY of 97.30% from PET textiles [85]. The method involves the initial synthesis of PET oligomer from PET fibers in a microwave reactor, followed by a hydrothermal reaction (at 260 °C for 24 h) of PET oligomer.

Silk, a natural biomaterial with rich nitrogen, is widely used to produce carbon materials [115,116]. CDs with nitrogen doping attracted attention because of their improved optical and electrical properties [117]. Waste silk cloth has been utilized as a carbon source to prepare CDs in an acid solution using a hydrothermal method (at 250 °C for 5 h), resulting in CDs with sizes ranging from 2.2 to 6.1 nm and a QY of 19.1% [88].

Table 4 illustrates that the hydrothermal method is the most widely reported to prepare CDs from waste cotton and wastepaper. The CDs derived from wastepaper (4.5 nm) had similar particle sizes to those derived from cellulose (4.2 nm) via a hydrothermal process at 180 °C. However, their QY shows a significant difference, standing at 10.8% and 21.7%, respectively. Burning is another method of making CDs from wastepaper. Water-soluble fluorescent CDs with a QY of 9.3% and a size distribution of 2–5 nm were obtained by simply incinerating wastepaper [80].

**Table 4:** CDs prepared from waste textiles and wastepaper and their corresponding properties.

Method	Carbon Precursor	Conditions	QY (%)	Size (nm)	Ref.
Hydrothermal method	Wastepaper	150–200 °C for 10 h	10.80	150 °C: 4.0–12.0 180 °C: 3.0–7.0 200 °C: 2.0–5.0	[79]
	Kraft softwood pulp	240 °C for 4 h	NA	Diameter: 2–6 Length: 40–60	[118]
	Carbon paper	180 °C for 8 h	∼5.1	4.8	[81]
	Wastepaper	210 °C for 12 h	10–27	2.6 to 4.4	[82]
	Wastepaper	220 °C for 15 h	20	2–4	[83]
	Degrease cotton (Human waste)	200 °C for 13 h	10.20	2–4	[84]
	Waste PET textiles	260 °C for 24 h	97.30	1.6–4.6	[85]
	Absorbent cotton	200 °C for 15 h	NA	1.4–5.6	[86]
	Terylene waste	260 °C for 18 h	49.36	2.5–7.0	[87]
	Waste silk cloth	250 °C for 5 h	19.10	2.2 ± 6.1	[88]
	Eucalyptus fibers	120 °C, 140 °C, 160 °C, and 180 °C for 24 h	NA	1.5–4.0	[89]
	Cellulose	180 °C for 72 h	21.7	4.2	[119]
	Cellulose	210 °C for 14 h	32.3	5.45	[120]
	Cellulose	200 °C for 12 h	2.9–18.3	2.11–8.72	[121]
	Microcrystalline Cellulose	240 °C for 12 h	54	0.5–6.5	[122]
Burn	Wastepaper	Burn	9.3	2–5	[80]

Note: NCC/CDs: nanocrystalline cellulose/carbon dots.

Similar to waste plastics, textiles and paper are solid waste materials and require pre-treatment prior to the preparation of CDs. Chemical pre-treatments can remove impurities from waste textiles. For example, waste PET fibers were converted to PET oligomers through glycolysis pre-treatment using ethylene glycol and zinc acetate dehydrates and subsequent microwave reactions. The resulting CDs have high QYs of 49.36% and 97.30%, with relatively small average sizes of 4.3 nm and 2.8 nm, respectively [85,87]. Various chemical methods have been reported to remove lignin from eucalyptus fibers, obtaining a refined cellulose fraction. This process often involves four sequential heat treatments with acetic acid and sodium chloride to remove lignin, followed by heating in the presence of potassium oxychloride to eliminate hemicellulose. The obtained N-CDs had an average size of 2.46 nm [89]. The high QY generated from PET-based waste was also confirmed in Table 4, where PET textiles were used as the precursor, and the QY could reach 97.3%. PET-CDs were prepared using a hydrothermal method with urea and homophthalic acid as co-precursors. The obtained CDs were not only successfully doped with nitrogen in the form of pyrrole N structures but also covered with -NH_2_ and abundant oxygen-containing functional groups [85].

Doping heteroatoms into CDs is an effective way to enhance QY. The most common doping element is nitrogen, which is generally introduced using N-containing small molecules, such as p-phenylenediamine, urea, ethylenediamine, and ammonia water as co-precursors [112,123,124,125]. Sulphur and phosphorus have also been co-doped with nitrogen by adding concentrated phosphoric and sulphuric acids, respectively [126,127,128,129]. These non-waste precursors help promote the hydrolysis and carbonization reactions and introduce heteroatom doping and surface functional groups, aiming to enhance the QY and induce specific interactions with analytes for sensing applications.

### 2.4. Other Wastes

**Figure 5:**
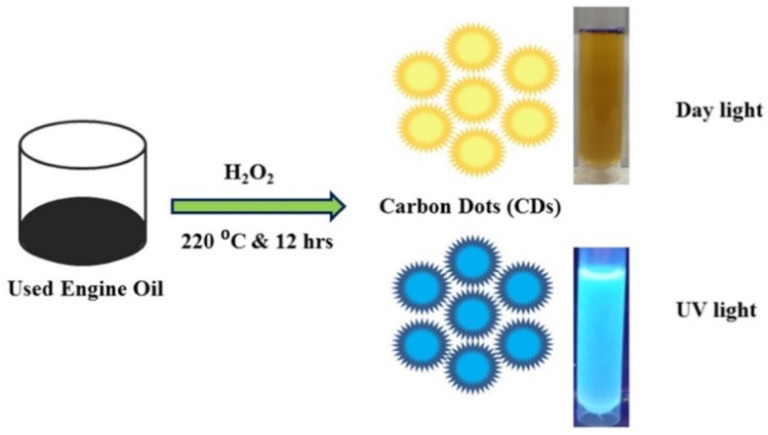
Waste-engine-oil-derived CDs. Reused with permission [92]. Copyright 2022 Elsevier.

Recently, sewage sludge [41], cigarette filters [90], and waste engine oil [92] have emerged as novel CD precursors. The pre-treatment approaches for converting these wastes into CDs depend on the composition of the materials. As urbanization continues, the volume of urban sludge composed of carbon-based substances increases rapidly. The untreated and inadequately treated sewage poses a significant threat to human life [130,131,132]. Sewage management technologies include various methods, such as landfill disposal, land spreading, anaerobic digestion, thermochemical processes, and integration into building materials [91,133]. Hu et al. reported the conversion of sewage sludge into useful CDs. This process involves pre-treatment through drying and grounding, followed by microwave irradiation. The resulting CDs had an average size of 4.0 nm with a high QY of 21.7% and could be used for fluorescent sensing applications, particularly for para-nitrophenol detection [41]. Waste engine oil was transformed into CDs using a direct hydrothermal method (220 °C for 12 h). The CDs showed a high QY of 11.4% and a size distribution of 2–10 nm with excellent detection selectivity and sensitivity towards Fe^3+^ (Figure 5) [92].

## 3. Applications of CDs Derived from Non-Biomass Waste

CDs derived from non-biomass waste have similar favorable properties, such as low toxicity, biocompatibility, high photostability, and fluorescence. These waste-derived CDs find applications in sensing, information encryption, LEDs, solar cells, and growth promotion.

### 3.1. Sensing

Table 5 summarizes the pre-treatment methods, synthesis techniques, and the corresponding sensing performance of reported CDs derived from non-biomass waste sources. The most common method to prepare CDs for sensing applications is the hydrothermal method, conducted at temperatures from 120 °C to 260 °C. Prior to this, the waste was subjected to various pre-treatment processes, such as purifying, grinding, sieving, drying, nitration, pyrolysis, oxidation, and microwave alcoholysis. CDs derived from non-biomass waste have been primarily utilized for the detection of heavy metal ions, followed by small molecules, pH, and bacteria. In Table 5, the fluorescence properties of CDs were used for almost all sensors, and one exception is that the impedance response of CDs derived from bike pollutant soot was used to detect relative humidity [41]. The fluorescence sensing behavior is dominated by turn-off detection, which involves the intensity quenching upon interacting with the analytes. The quenching mechanisms include static or dynamic quenching. In static quenching, a non-emissive complex is formed between the CDs and the analyte, causing a previously emissive state to return to the ground state without an emission. Dynamic quenching, often referred to as collisional quenching, occurs because of the collisions or close contact between the analyte and the excited CDs, which result in an energy transfer without an emission. Dynamic quenching includes several mechanisms, such as photo-induced electron transfer, Förster resonance energy transfer, surface energy transfer, and inner filter effect (IFE) [134].

Heavy metals, such as Fe^3+^, Hg^2+^, Cu^2+^, Cr^4+^, and Au^3+^, can accumulate in the eco-systems, causing harmful effects on the environment and living organisms [135]. CDs derived from various non-biomass waste categories using the hydrothermal method have been reported for highly selective and sensitive heavy metal sensing. For example, CDs prepared from medical masks, waste engine oil, and waste PET were utilized for Fe^3+^ quantitation with linear ranges of 1–300 μM, 0.5–400 μM, and 0.6–3.3 μM and limits of detections (LODs) of 0.11 μM, 0.21 μM, and 0.055 μM, respectively. The average size of CDs ranged from 3.7 nm to 6 nm. CDs with average sizes from 2.5 nm to 6 nm, derived from PET, polyolefin, and cotton using the hydrothermal approach, have enabled sensitive and selective detections of Pb^2+^, Cu^2+^, and Cr^4+^ with LODs of 21 nM, 6.33 nM, and 0.12 μg/mL, respectively. The hydroxyl and carboxyl groups on the surface of the CDs interact with heavy metal ions, resulting in static or IFE fluorescence quenching [71,75,83,92]. Doping with nitrogen is a common strategy to enhance the fluorescent properties and increase quenching probabilities of CDs because of the presence of functional groups such as amine, hydroxyl, carbonyl, nitryl, and alkene [49,117]. Additionally, N-CDs derived from candle soot have an average size between 2 nm to 5 nm and have been used for quantifying Fe^3+^ and Hg^2+^ in water with a similar linear range of 20–50 μM. The LODs for Fe^3+^ and Hg^2+^ are 10 nM and 50 nM, respectively. The fluorescence generated by electron transfer of N-CDs is captured by empty ‘d’ orbital of Fe^3+^ and Hg^2+^, leading to a PET quenching mechanism [49]. N-CDs with a QY of 20% and an average size of 4.0 ± 1.2 nm were synthesized from waste-expanded polystyrene (EPS) using the one-step solvothermal method, exhibiting selectivity for Au^3+^ quantitation with an LOD of 53 nM [70]. PU, rich in nitrogen atoms, is an ideal candidate for synthesizing highly photoluminescent CDs with enhanced QYs. N-CDs derived from waste white PU foam had diameters ranging from 5 nm to 8 nm and a relatively high QY of 33%. The CDs could detect Ag^+^ with an LOD of 2.8 μM. The quenching effect is attributed to static quenching due to a strong interaction between the S-doped surface of CDs and Ag^+^ [73].

In addition to heavy metals, CDs from non-biomass sources have been employed to detect small molecules, such as para-nitrophenol [41], tetracycline [90], trinitrotoluene [81], tartrazine [51], pesticides [83], water in organic solvent [45,58], and cholesterol [42]. The CDs prepared from PET waste showed a highly selective and sensitive detection of ferric ion (Fe^3+^) through a quenching effect, and the fluorescence could be restored specifically with pyrophosphate anion (PPi), rendering the CDs/Fe^3+^ sensor promising for PPi detection [75]. The static quenching mechanism of CDs was caused by Fe^3+^ due to the formation of nonfluorescent CD-Fe^3+^ complexes. Compared with CDs, PPi possessed a stronger affinity toward Fe^3+^ to generate PPi-Fe^3+^ complexes, thus releasing CDs and recovering the fluorescence. Similarly, the burning ash of the wastepaper was used as a carbon source to synthesize CDs. The fluorescence of obtained CDs could be turned off by Fe^3+^, which was derived from Fe^2+^ oxidized by H_2_O_2_. Organophosphorus pesticides effectively inhibited the production of H_2_O_2_ by destroying the acetylcholinesterase activity, so the fluorescence of CDs was turned on in the presence of organophosphorus pesticides [82]. N-CDs synthesized from carbon paper and waste PET derived using solvothermal methods have small average sizes of 4.8 nm and 1.93 nm, respectively. They have been used for the quantitation of tetracycline and trinitrotoluene in both water and in organic solvents. Furthermore, CDs were prepared from single-use plastic waste, such as plastic polybags, cups, and bottles, via a hydrothermal method (at 200 °C for 5 h) with high QY of 60%, 65%, and 69%, respectively. They demonstrated the ability to effectively sense *E. coli* with an LOD of 108 CFU/mL [67]. Empty PET bottles were pre-treated using a microwave reactor, followed by crushing into powder using a pulverizer. Nitrogen- and phosphorus-doped CDs with spherical structures and an average particle size of 2.8 nm have been applied for pH sensing in the range of 2.3 to 12.3 [77].

### 3.2. Information Encryption

CDs are considered one of the most promising candidates for information encryption due to their polychromatic emission, a wide array of luminous categories, and stable physicochemical properties [136]. These versatile materials have been successfully synthetized from wastepaper using various solvents, such as deionized water, ethanol, and 2-propanol, using a hydrothermal method at 210 °C. The obtained CDs with average sizes from 2.6 nm to 4.4 nm and QYs of 12%, 27%, and 10% showed emission colors spanning from blue to yellow and have found applications as anti-counterfeiting ink for fluorescent flexible films [82].

### 3.3. LEDs

LEDs, as solid-state devices, have a crucial role in relieving the energy crisis. CDs have made significant contributions to recent advancements in LEDs because of their excellent photoluminescence and high stability [137]. Table 6 summarizes the LED applications of CDs derived from non-biomass wastes. CDs prepared from waste PET, non-degradable products and waste EPS prepared using solvothermal approaches could have multiple colors with particle sizes from 2.0 nm to 4.5 nm. Waste-PET-derived CDs also exhibited a range of colors, including colorless, white, yellow, blue–green, and brown [57,58,69,77,85]. Biohazardous products, such as PPE plastic waste, used disposable gloves, face shields, syringes, and food storage containers and bottles, were utilized to prepare CDs using a pyrolytic method. The resulting N-CDs emitted white light and possessed a high QY of 41% [73]. Furthermore, CDs with an average size of 2.1 nm were derived from waste toner powder via microwave irradiation. These CDs emitted yellow light at 557 nm under 300 nm excitation and had been used in LEDs [55].

### 3.4. Solar Cells

Solar energy conversion is pivotal in addressing climate change [138]. The transformation of non-biomass waste into CDs can reduce pollution, and their subsequent utilization in solar energy conversion holds the potential to yield substantial societal, economic, and environmental benefits. A series of CDs have been successfully synthesized from absorbent cotton using a one-pot hydrothermal method. By introducing different dopants, such as carbamide, thiourea, and 1,3-diaminopropane, the average particle sizes were significantly reduced from 24.2 nm to 1.7 nm, 5.6 nm, and 1.4 nm, respectively. The 1,3-diaminopropane-doped CDs showed the highest power conversion efficiency (PCE) of 0.527%, which was 299% higher than that achieved without dopant (0.176%) [86].

### 3.5. Plant Growth Promotion

CDs, as a new type of carbon material have demonstrated their potential to boost plant growth [139,140]. For instance, PET was thermally treated at 400 °C for 2 h and crushed into a fine powder using ball milling, followed by a subsequent hydrothermal process (110 °C for 15 h) in the presence of H_2_O_2_ solution. When applied at concentrations of 0.25 mg/mL to 2 mg/mL, these CDs with an average size of 2.5 ± 0.5 nm could enhance the development of shoots and roots during germination and growth of pea (*Pisum sativum*). It is believed that the interaction between CDs and pea seeds promotes growth [62]. Similarly, CDs prepared from various plastic products via direct thermal treatment at high temperatures (800 °C for 1 h) promoted the growth of C. arietinum seeds within the concentration range of 0.1 mg/mL to 0.5 mg/mL [40]. However, the specific mechanism remains unclear. Furthermore, carbon nanomaterials with sizes ranging from 20 nm to 100 nm were synthesized from oil fly ash using a high-energy ball milling method. These CDs had been used in the treatment of *Phaseolus vulgaris* L. and *Cicer arietinum* L. plants [43].

## 4. Conclusions

The rising concerns about air and water pollution, land degradation, and the economic cost associated with increasing waste have garnered significant social concerns. An effective approach to address these issues is to convert waste into CDs for high-end applications. Considering that CDs derived from biomass waste have been widely reported, this review focuses on non-biomass waste, especially the related preparation methods, properties, and applications. Selecting the most suitable methods for synthesizing CDs from non-biomass waste requires careful consideration of the properties of the waste materials. Compared to CDs derived from chemicals, the complexity of the raw material composition presents a significant challenge. Pre-treatments, which may involve physical and chemical methods, are often essential to remove impurities and convert solid waste into powder forms suitable for CDs synthesis. However, the complexity of these procedures, the use of highly toxic chemicals, and the requirement for high temperatures and pressure may limit the applicability of these methods. CDs obtained from non-biomass waste have found applications in sensing, information encryption, LEDs, solar cells, and plant growth promotion.

The conversion of non-biomass waste into CDs is still in the early stages. The mechanism for enhancing QYs remains unclear. Industrial-scale production of CDs from non-biomass waste materials represents an efficient way of value-adding and reducing environmental impact. Challenges in this research field include:

(1) Expanding the range of non-biomass waste materials as carbon precursors for CDs synthesis.
(2) Simplifying pre-treatment procedures by reducing the use of toxic chemicals, lowering temperatures, and decreasing pressure.
(3) Exploring methods to enhance the properties of CDs, especially QY.
(4) Developing techniques to synthesize CDs from mixed non-biomass waste sources.
(5) Broadening the scope of CD applications from non-biomass waste.

Combining waste management strategies with CD synthesis technology offers an effective approach to addressing these technical challenges. Analyzing the components within the non-biomass waste and referencing methods used for precursors with similar chemical structures can be highly beneficial in developing a new route to convert non-biomass waste into CDs. Various synthetic approaches for CDs from chemicals encompass top-down methods, such as ball milling, laser ablation, arc discharge, chemical oxidation, electrochemical methods, micro-fluidization, and plasma approaches, as well as bottom-up approaches, such as pyrolytic methods, template, microwave-assisted, ultrasonic, hydrothermal/solvothermal, and chemical oxidation. Some of these methods have been used to convert non-bio waste to CDs, including reflux, hydrothermal, ball milling, ultrasonic irradiation, pyrolysis, and microwave-assisted methods. The applicability of the other methods warrants further study. In the experimental design, the selection of non-toxic, cost-effective, and environmentally friendly chemicals and methods is crucial to minimize any potential environmental pollution. The guiding principle should be followed when designing CDs from non-biomass waste. Surface functionalization and the doping of chemical heteroatoms have been designed to enhance the optical, electrical, and chemical properties of CDs, thereby expanding their potential applications.

## Figures and Tables

**Table 1:** Summary of the foci of past reviews on waste-derived CDs.

Title	Foci	Ref.
A Review of Carbon Dots Produced from Biomass Wastes	Methods and applications of CDs from biomass waste. Advantages and disadvantages of CDs from biomass waste. Major influencing factors on photoluminescence characteristics.	[17]
Recent Trends in the use of Green Sources for Carbon Dot Synthesis—A short review	Synthesis of CDs from green sources including biomass waste and non-biomass waste.	[18]
A Review on Multifunctional Carbon-Dots Synthesized from Biomass Waste: Design/Fabrication, Characterization and Applications	Methods and applications of CDs from biomass waste. Structure analysis, physical, and chemical properties of CDs. The factors affecting the bandgap formation mechanisms of the CDs produced by hydrothermal methods.	[19]
Carbon Quantum Dots Synthesis from Waste and By-Products: Perspectives and Challenges	Potentials, advantages, and challenges in synthesizing CDs from waste after comparing the quantum yield.	[20]
Food Waste as a Carbon Source in Carbon Quantum Dots Technology and their Applications in Food Safety Detection	Approaches, characterizations, and applications of CDs from food wastes. Applications on food quality and safety detection, especially on sensing food additives and heavy metal ions.	[21]
Green Carbon Dots with Multifaceted Applications—Waste to Wealth Strategy	Synthesis routes, fluorescent properties and mechanisms, and applications of CDs from wastes with a focus on hydrothermal approach.	[14]
Recent Advances of Biomass Carbon Dots on Syntheses, Characterization, Luminescence Mechanism, and Sensing Applications	Synthesis and properties improvement methods for CDs from biomass. Characterization of the structure, composition of biomass-derived CDs, and the regulation of fluorescence color. Luminescence mechanism and sensing applications.	[22]
Sustainable Synthesis of Multifunctional Carbon Dots using Biomass and their Applications: A mini review	Synthesis methods, especially hydrothermal methods, applications, and characterizations of CDs from plant sources. Separation technologies.	[23]
Carbon Dots based on Natural Resources: Synthesis and Applications in Sensors	Synthesis of CDs from biomass resources and their sensing applications.	[24]
Biomass-Based Carbon Dots: Current Development and Future Perspectives	Advantages and disadvantages on synthesis, properties, and applications of CDs from biomass waste and chemicals.	[25]
New Insight into the Engineering of Green Carbon Dots: Possible Applications in Emerging Cancer Theragnostic	Synthesis, physicochemical properties, and possible applications of CDs from natural sources.	[26]
Green Synthesis of Carbon Quantum Dots and their Environmental Applications	Synthesis and physicochemical properties and stability of CDs. Applications in wastewater treatment and biomedical fields.	[27]
Sustainable Development of Carbon Nanodots Technology: Natural Products as a Carbon Source and Applications to Food Safety	Synthesis of CDs from food and food waste. Application of photoluminescent CDs in food safety.	[28]
Biomass-Derived Carbon Dots and Their Applications	Simple synthesis routes and specific optical properties of CDs from biomass. Applications in biosensing, bioimaging, optoelectronics, and catalysis.	[29]
Plastic Waste-Derived Carbon Dots: Insights of Recycling Valuable Materials Towards Environmental Sustainability	Synthesis routes, characterizations, and potential applications of CDs from plastic waste.	[30]
The Role of Fluorescent Carbon Dots in the Fate of Plastic Waste	Approaches, properties, and applications of CDs from plastic waste. The role of CDs in the fate of plastic waste.	[31]

**Table 2:** CDs prepared from waste ash and coal and their corresponding properties.

Method	Carbon Precursor	Conditions	Size (nm)	QY (%)	Ref.
Ball Milling	Oil fly ash	25 Hz and 400 rpm for 45 h in the air	<35	NA	[43]
	Oil fly ash	25 Hz for 45 h in acetic acid	<100	NA	[44]
Burn	Cigarette Smoking	In the air	4.5–7.0	NA	[50]
	Ethanol, n-Butanol, Domestic candle, and Benzene	In the air	2.0–4.0	NA	[32]
Chemical Oxidation	Pollutant diesel soot	10 h	20–50	1.9	[42]
	Vehicle exhaust waste soot	100 °C for 12 h	2.2−4.6	3	[51]
	Candle soot	140 °C for 12 h	112	0.5	[53]
	Waste candle soot	110 °C for 6 h	2.0–5.0	NA	[46]
	Fullerene carbon soot	80–120 °C for 12–36 h	2.0–3.0	3–5	[54]
	Kerosene fuel soot	100 °C for 12 h	1.0–7.0	NA	[52]
	Candle soot	80 °C for 6 h	2.0–5.0	NA	[49]
	Candles	20 h	10–45	NA	[48]
	Coal-T20	Ice-cold condition for 6 h	2.0–5.0	3	[95]
	Coal-NK	Ice-cold condition for 6 h	10–30	4	[95]
	Coal- T60	Ice-cold condition for 6 h	1.0–6.0	8	[95]
	Coal-NG	Ice-cold condition for 6 h	1.0–4.0	14	[95]
	Gondwana coal, Damodar Coal, Tertiary Indian coal	2 h	4.8–14.0	NA	[96]
	Pennsylvania anthracite, and Kentucky bituminous coals	Ice-cold condition for 5–6 h	2.0–12.0	4–53	[97]
Soxhlet-Purification	Diesel soot	In acetone	20–30	~8	[39]
Hydrothermal Method	Bike Pollutant Soot	160 °C for 10 h	1–10	NA	[45]
Microwave pyrolysis	Red toner powder	350 W for 30 s	1–4	9.2 for internal and 8.4 for external efficiency	[55]

**Table 3:** CDs prepared from waste plastics and their corresponding properties.

Method	Carbon Precursor	Conditions	QY (%)	Size (nm)	Ref.
Hydrothermal approach	Waste PET bottles	180 °C for 12 h in diethylenetriamine (DETA) with H_2_O_2_	9.1	3.9–12.9	[56]
	Waste PET bottles	260 °C for 12 h in ammonia water	87.36	1.1–3.1	[57]
	Waste PET bottles	260 °C for 36 h	48.16	1.6–2.9	[58]
	PLA polymeric waste	240 °C for 4 h in ultrapure water	NA	2.99 ± 0.57	[59]
	PS plastics	180 °C for 8 h with HNO_3_ and ethylenediamine	NA	2.66–5.18	[61]
	Waste PET bottles	110 °C for 15 h in H_2_O_2_	NA	1.3–4.0	[62]
	PE plastic bags PP surgical masks	180 °C for 12 h in HNO_3_	14 16	1.0–8.0	[63]
	Waste PET bottles	200 °C for 8 h in deionized water	31.81	3.0–10.0	[65]
	Plastic polybags Cups Bottles	300 °C for 2 h of thermal calcination and 200 °C for 5 h of hydrothermal treatment in deionized water	60–69	NA	[67]
	Waste PET bottles	350 °C for 2 h in air and hydrothermal treatment at 180 °C for 12 h in H_2_O_2_ solution.	5.2	3.0–10.0	[68]
	Waste expanded PS Foam	200 °C for 5 h in HNO_3_	W-CDs *: 5.2, Y-CDs *: 3.4% O-CDs *: 3.1%	W-CDs *: 4.5, Y-CDs *: 3.5, O-CDs *: 2.3	[69]
	Waste medical masks	200 °C for 10 h in deionized water	NA	1.0–6.0	[71]
	Waste polyolefins	120 °C for 12 h in HNO_3_ and H_2_SO_4_	4.84	1.5–3.5	[72]
	Waste PET bottles	180 °C for 12 h in H_2_O_2_	5.2	3.0–10.0	[76]
	Waste PET bottles	260 °C for 24 h	14.2	1.8–4.6	[77]
Pyrolysis method	Waste plastic cups	350 °C for 2 h	59	<10	[66]
	Waste PET bottles	800 °C for 1h	NA	2000–8000	[40]
	PU foam	200, 250, and 300 °C for 2, 4, and 6 h	33	5.0–8.0	[74]
	HDPE/LDPE, PET, PS, PVC, PP	800 °C for 1 h	NA	NA	[75]

* Note: W-CD: white CDs; Y-CD: yellow CDs; O-CD: orange CDs; PET: polyester; PLA: polylactide; PS: polystyrene; PE: polyethylene; PP: polypropylene; PU: polyurethane; HDPE: High-Density Polyethylene; LDPE: Low-Density Polyethylene; PVC: polyvinylchloride; NA: not available.

**Table 5:** Sensing applications of CDs derived from non-biomass waste.

Carbon Precursor	Pre-Treatment	Method	Analyte	Limits of Detection (LOD)	Linear Range	Ref.
Harmful diesel soot	Magnetically purified	Soxhlet-purification with acetone	Fe^3+^ and Hg^2+^	Fe^3+^: ~352 nM Hg^2+^: ~898 nM	NA	[39]
Candle soot	HNO_3_ and ethanol treatment	Stirring at 80 °C for 6 h with ethylene diamine and sodium lauryl sulphate (SDS)	Hg^2+^ and Fe^3+^	Fe^3+^: 10 nM Hg^2+^: 50 nM	Fe^3+^: 20–50 μM Hg^2+^: 20–50 μM	[49]
PS plastics	Nitration	Solvothermal treatment at 180 °C for 8 h	Hg^2+^, Fe^3+^, and GSH	NA	Fe^3+^: 0.25–10 μM Hg^2+^: 0.5–20 μM GSH: 1–50 μM	[61]
Waste medical masks	NA	Hydrothermal treatment at 200 °C for 10 h	Na_2_S_2_O_4_ and Fe^3+^	Na_2_S_2_O_4_: 19.44 μM Fe^3+^: 0.11 μM	Na_2_S_2_O_4_: 0.1–5 mM Fe^3+^: 1–300 μM	[71]
Waste engine oil	Filtration process by filter paper	Hydrothermal treatment at 200 °C for 12 h	Fe^3+^	0.055 μM	0.6–3.3 μM	[92]
Waste PET bottles	Pyrolysis at 350 °C for 2 h in air	Hydrothermal treatment at 180 °C for 12 h	Fe^3+^ and pyrophosphate ions	Fe^3+^: 0.21 μM pyrophosphate: 0.86 μM	Fe^3+^: 0.5–400 μM pyrophosphate: 2–600 μM	[76]
Waste PET bottles	Shredding and air oxidation at 350 °C for 2 h.	Hydrothermal treatment at 170 °C for 8 h	Pb^2+^	21 nM	0–2 μM	[68]
Waste expanded PS	NA	One-step solvothermal method at 150 °C for 8 h	Au^3+^	53 nM	0–18 μM	[70]
White PU foam	Crushed	Pyrolysis at 200, 250, and 300 °C for 2, 4, and 6 h in H_2_SO_4_	Ag^+^	2.8 μM	NA	[74]
Waste PO	Pyrolysis by ultrasonic and chemical oxidation approach at 700 W for 2 h.	Hydrothermal method at 120 °C for 12 h	Cu^2+^	6.33 nM	1–8.0 μM	[72]
Degrease cotton	NA	One-pot hydrothermal method at 200 °C for 13 h	Cr^4+^	0.12 μg/mL	1–6 mmol/L	[84]
Waste plastic cups	NA	Simple thermal calcination at 350 °C for 2 h	Sulphite anion	0.34 μM	0.001–50 μm	[66]
Sewage sludge	Dried and grounded into fine powder	Microwave-assisted heating with 700 W for 30 min.	Para-Nitrophenol	0.069 μM	0.2–20 μM	[41]
Cigarette filters	Cut and dried in an oven at 80 °C for 1 h	One-pot hydrothermal method at 240 °C for 15 h	Tetracycline	0.06 μM	0–80 μM	[90]
Carbon paper	Burn	Hydrothermal route at 180 °C for 8 h	Trinitrotoluene	32.7 nM	4.4 nM–26.4 μM	[81]
Vehicle exhaust waste soot	NA	One-pot acid reflexion method with nitric acid at 100 °C for 12 h	Tartrazine	26 nM	0.1 to 0.5 μM	[51]
Wastepaper	NA	Hydrothermal method at 220 °C for 15 h	Organophosphorus pesticides	3 ng/mL	0.01–1.0 μg/mL	[83]
PET waste bottles	Microwave alcoholysis with 540 W for 20 min followed by crushing into powder	Solvothermal method at 260 °C for 36 h	Water in organic solvent	0.00001%	NA	[58]
Pollutant diesel soot	Purified via Soxhlet extraction method with different organic solvents	Chemical oxidation method refluxed 10 h	Cholesterol and *E. coli*	NA	NA	[42]
Single-use plastic waste such as plastic polybags, cups, and bottles	Calcination at 300 °C for 2 h.	Hydrothermal treatment at 200 °C for 5 h,	*E. coli*	108 CFU/mL	NA	[67]
Waste PET bottles	Microwave alcoholysis with 540 W for 20 min followed by crashing into powder	Solvothermal method at 260 °C for 24 h	pH	NA	NA	[77]
Bike pollutant soot	Ground for 1 h and sieved using 15 mm sieving paper	Hydrothermal treatment at 160 °C for 10 h	Humidity	NA	NA	[45]

Note: NA: not available.

**Table 6:** LED applications of CDs derived from non-biomass waste.

Carbon Precursor	Method	Emission Peak (nm)	Light Color	Size (nm)	QY (%)	Ref.
Waste PET bottles	Hydrothermal	485	Colorless to brown	2.0	87.36	[57]
Waste PET bottles	Solvothermal	360 470	Yellow light warm light	2.3	48.16	[58]
Waste expanded PS	Solvothermal	470 530 630	White Yellow Orange	4.5 3.5 2.3	5.2 3.4 3.1	[69]
PPE plastic waste, used disposable gloves, face shields, syringes, and food storage containers and bottles	Pyrolytic	436 495	White light	NA	41	[73]
Waste PET bottles	Solvothermal	460	White light	2.8	14.2	[77]
Waste PET textiles	Hydrothermal	485	Blue-green light	2.8	97.3	[85]
Wasted toner powder	Microwave irradiation	557	Yellow light	2.1	9.2 for internal and 8.4 for external efficiency	[55]

## References

[1] Parmesan C., Morecroft M.D., Trisurat Y. (2022). Climate Change 2022: Impacts, Adaptation and Vulnerability. Ph.D. Thesis.

[2] Gautam M., Agrawal M. (2021). Greenhouse gas emissions from municipal solid waste management: A review of global scenario. Carbon Footprint Case Studies: Municipal Solid Waste Management, Sustainable Road Transport and Carbon Sequestration.

[3] Maria C., Góis J., Leitão A. (2020). Challenges and perspectives of greenhouse gases emissions from municipal solid waste management in Angola. Energy Rep..

[4] Yu K.H., Zhang Y., Li D., Montenegro-Marin C.E., Kumar P.M. (2021). Environmental planning based on reduce, reuse, recycle and recover using artificial intelligence. Environ. Impact Assess. Rev..

[5] Zorpas A.A. (2020). Strategy development in the framework of waste management. Sci. Total Environ..

[6] Kumar R., Verma A., Shome A., Sinha R., Sinha S., Jha P.K., Kumar R., Kumar P., Shubham, Das S. (2021). Impacts of plastic pollution on ecosystem services, sustainable development goals, and need to focus on circular economy and policy interventions. Sustainability.

[7] Shams M., Alam I., Mahbub M.S. (2021). Plastic pollution during COVID-19: Plastic waste directives and its long-term impact on the environment. Environ. Adv..

[8] Moazzem S., Wang L., Daver F., Crossin E. (2021). Environmental impact of discarded apparel landfilling and recycling. Resour. Conserv. Recycl..

[9] Moazzem S., Crossin E., Daver F., Wang L. (2021). Environmental impact of apparel supply chain and textile products. Environ. Dev. Sustain..

[10] Tomaras J. (2021). Waste Management and Recycling. https://www.aph.gov.au/About_Parliament/Parliamentary_Departments/Parliamentary_Library/pubs/rp/BudgetReview202021/WasteManagementRecycling.

[11] Joe Pickin C.W., O’Farrell K., Stovell L., Nyunt P., Guazzo S., Lin Y., Caggiati-Shortell G., Chakma P., Edwards C., Lindley B. (2022). National Waste Report 2022.

[12] Tu L., Li Q., Qiu S., Li M., Shin J., Wu P., Singh N., Li J., Ding Q., Hu C. (2023). Recent developments in carbon dots: A biomedical application perspective. R. Soc. Chem. J..

[13] Tran N.-A., Hien N.T., Hoang N.M., Dang H.-L.T., Van Quy T., Hanh N.T., Vu N.H., Dao V.-D. (2023). Carbon dots in environmental treatment and protection applications. Desalination.

[14] Shahraki H.S., Ahmad A., Bushra R. (2022). Green carbon dots with multifaceted applications—Waste to wealth strategy. FlatChem.

[15] Truskewycz A., Yin H., Halberg N., Lai D.T., Ball A.S., Truong V.K., Rybicka A.M., Cole I. (2022). Carbon dot therapeutic platforms: Administration, distribution, metabolism, excretion, toxicity, and therapeutic potential. Small.

[16] Houshyar S., Yin H., Pope L., Zizhou R., Dekiwadia C., Hill-Yardin E.L., Yeung J.M., John S., Fox K., Tran N. (2023). Smart suture with iodine contrasting nanoparticles for computed tomography. OpenNano.

[17] Kang C., Huang Y., Yang H., Yan X.F., Chen Z.P. (2020). A review of carbon dots produced from biomass wastes. Nanomaterials.

[18] Kurian M., Paul A. (2021). Recent trends in the use of green sources for carbon dot synthesis—A short review. Carbon Trends.

[19] Khairol Anuar N.K., Tan H.L., Lim Y.P., So’aib M.S., Abu Bakar N.F. (2021). A review on multifunctional carbon-dots synthesized from biomass waste: Design/fabrication, characterization and applications. Front. Energy Res..

[20] de Oliveira B.P., da Silva Abreu F.O.M. (2021). Carbon quantum dots synthesis from waste and by-products: Perspectives and challenges. Mater. Lett..

[21] Fan H., Zhang M., Bhandari B., Yang C.-h. (2020). Food waste as a carbon source in carbon quantum dots technology and their applications in food safety detection. Trends Food Sci. Technol..

[22] Lou Y., Hao X., Liao L., Zhang K., Chen S., Li Z., Ou J., Qin A., Li Z. (2021). Recent advances of biomass carbon dots on syntheses, characterization, luminescence mechanism, and sensing applications. Nano Sel..

[23] Perumal S., Atchudan R., Edison T.N.J.I., Lee Y.R. (2021). Sustainable synthesis of multifunctional carbon dots using biomass and their applications: A mini-review. J. Environ. Chem. Eng..

[24] Lin X., Xiong M., Zhang J., He C., Ma X., Zhang H., Kuang Y., Yang M., Huang Q. (2021). Carbon dots based on natural resources: Synthesis and applications in sensors. Microchem. J..

[25] Wareing T.C., Gentile P., Phan A.N. (2021). Biomass-based carbon dots: Current development and future perspectives. ACS Nano.

[26] Radnia F., Mohajeri N., Zarghami N. (2020). New insight into the engineering of green carbon dots: Possible applications in emerging cancer theranostics. Talanta.

[27] Manikandan V., Lee N.Y. (2022). Green synthesis of carbon quantum dots and their environmental applications. Environ. Res..

[28] Huang C.-C., Hung Y.-S., Weng Y.-M., Chen W., Lai Y.-S. (2019). Sustainable development of carbon nanodots technology: Natural products as a carbon source and applications to food safety. Trends Food Sci. Technol..

[29] Meng W., Bai X., Wang B., Liu Z., Lu S., Yang B. (2019). Biomass-derived carbon dots and their applications. Energy Environ. Mater..

[30] Arpita, Kumar P., Kataria N., Narwal N., Kumar S., Kumar R., Khoo K.S., Show P.L. (2023). Plastic Waste-Derived Carbon Dots: Insights of Recycling Valuable Materials Towards Environmental Sustainability. Curr. Pollut. Rep..

[31] Hallaji Z., Bagheri Z., Ranjbar B. (2023). The role of fluorescent carbon dots in the fate of plastic waste. J. Environ. Chem. Eng..

[32] Zhang S., Zhang L., Huang L., Zheng G., Zhang P., Jin Y., Jiao Z., Sun X. (2019). Study on the fluorescence properties of carbon dots prepared via combustion process. J. Lumin..

[33] Chahal S., Macairan J.-R., Yousefi N., Tufenkji N., Naccache R. (2021). Green synthesis of carbon dots and their applications. RSC Adv..

[34] Hu S., Trinchi A., Atkin P., Cole I. (2015). Tunable photoluminescence across the entire visible spectrum from carbon dots excited by white light. Angew. Chem. Int. Ed..

[35] Kolanowska A., Dzido G., Krzywiecki M., Tomczyk M.M., Łukowiec D., Ruczka S., Boncel S. (2022). Carbon quantum dots from amino acids revisited: Survey of renewable precursors toward high quantum-yield blue and green fluorescence. ACS Omega.

[36] Zhu S., Tang S., Zhang J., Yang B. (2012). Control the size and surface chemistry of graphene for the rising fluorescent materials. Chem. Commun..

[37] Yan F., Sun Z., Zhang H., Sun X., Jiang Y., Bai Z. (2019). The fluorescence mechanism of carbon dots, and methods for tuning their emission color: A review. Microchim. Acta.

[38] Sk M.A., Ananthanarayanan A., Huang L., Lim K.H., Chen P. (2014). Revealing the tunable photoluminescence properties of graphene quantum dots. J. Mater. Chem. C.

[39] Kaushik J., Saini D., Singh R., Dubey P., Sonkar S.K. (2021). Surface adhered fluorescent carbon dots extracted from the harmful diesel soot for sensing Fe (III) and Hg (II) ions. New J. Chem..

[40] Mondal N.K., Singha P., Sen K., Mondal A., Debnath P., Mondal A., Mishra D. (2023). Waste plastics acts as a good growth promoter: A laboratory-based study. Res. Sq..

[41] Hu Y., Gao Z. (2020). Sewage sludge in microwave oven: A sustainable synthetic approach toward carbon dots for fluorescent sensing of para-Nitrophenol. J. Hazard. Mater..

[42] Tripathi K.M., Sonker A.K., Sonkar S.K., Sarkar S. (2014). Pollutant soot of diesel engine exhaust transformed to carbon dots for multicoloured imaging of *E. coli* and sensing cholesterol. RSC Adv..

[43] Alluqmani S.M., Alabdallah N.M. (2022). Preparation and application of nanostructured carbon from oil fly ash for growth promotion and improvement of agricultural crops with different doses. Sci. Rep..

[44] Alluqmani S.M., Loulou M., Ouerfelli J., Alshahrie A., Salah N. (2021). Annealing effect on structural and optical properties of nanostructured carbon of oil fly ash modified titania thin-film. Results Phys..

[45] Chaudhary P., Verma A., Mishra A., Yadav D., Pal K., Yadav B., Kumar E.R., Thapa K.B., Mishra S., Dwivedi D. (2022). Preparation of carbon quantum dots using bike pollutant soot: Evaluation of structural, optical and moisture sensing properties. Phys. E Low-Dimens. Syst. Nanostructures.

[46] Ganesan K., Hayagreevan C., Jeevagan A.J., Adinaveen T., Sophie P.L., Amalraj M., Bhuvaneshwari D. (2023). Candle soot derived carbon dots as potential corrosion inhibitor for stainless steel in HCl medium. J. Appl. Electrochem..

[47] Huang H., Cui Y., Liu M., Chen J., Wan Q., Wen Y., Deng F., Zhou N., Zhang X., Wei Y. (2018). A one-step ultrasonic irradiation assisted strategy for the preparation of polymer-functionalized carbon quantum dots and their biological imaging. J. Colloid Interface Sci..

[48] Li Y., Chen T., Ma Y. (2016). Nanosized carbon dots from organic matter and biomass. J. Wuhan Univ. Technol. Mater. Sci. Ed..

[49] Pankaj A., Tewari K., Singh S., Singh S.P. (2018). Waste candle soot derived nitrogen doped carbon dots based fluorescent sensor probe: An efficient and inexpensive route to determine Hg (II) and Fe (III) from water. J. Environ. Chem. Eng..

[50] Song Y., Lu F., Li H., Wang H., Zhang M., Liu Y., Kang Z. (2018). Degradable carbon dots from cigarette smoking with broad-spectrum antimicrobial activities against drug-resistant bacteria. ACS Appl. Bio Mater..

[51] Thulasi S., Kathiravan A., Asha Jhonsi M. (2020). Fluorescent carbon dots derived from vehicle exhaust soot and sensing of tartrazine in soft drinks. ACS Omega.

[52] Venkatesan S., Mariadoss A.J., Arunkumar K., Muthupandian A. (2019). Fuel waste to fluorescent carbon dots and its multifarious applications. Sens. Actuators B Chem..

[53] Wang Q., Zheng H., Long Y., Zhang L., Gao M., Bai W. (2011). Microwave–hydrothermal synthesis of fluorescent carbon dots from graphite oxide. Carbon.

[54] Zhang Q., Sun X., Ruan H., Yin K., Li H. (2017). Production of yellow-emitting carbon quantum dots from fullerene carbon soot. Sci. China Mater..

[55] Hong W.T., Moon B.K., Yang H.K. (2022). Microwave irradiation and color converting film application of carbon dots originated from wasted toner powder. Mater. Res. Bull..

[56] Chan K., Zinchenko A. (2022). Aminolysis-assisted hydrothermal conversion of waste PET plastic to N-doped carbon dots with markedly enhanced fluorescence. J. Environ. Chem. Eng..

[57] Wang R., Li S., Huang H., Liu B., Gao L., Qu M., Wei Y., Wei J. (2023). Preparation of carbon dots from PET waste by one-step hydrothermal method and its application in light blocking films and LEDs. J. Fluoresc..

[58] Ma G., Wang R., Zhang M., Dong Z., Zhang A., Qu M., Gao L., Wei Y., Wei J. (2023). Solvothermal preparation of nitrogen-doped carbon dots with PET waste as precursor and their application in LEDs and water detection. Spectrochim. Acta Part A Mol. Biomol. Spectrosc..

[59] Lauria A., Lizundia E. (2020). Luminescent carbon dots obtained from polymeric waste. J. Clean. Prod..

[60] Hu Y., Li M., Gao Z., Wang L., Zhang J. (2021). Waste polyethylene terephthalate derived carbon dots for separable production of 5-hydroxymethylfurfural at low temperature. Catal. Lett..

[61] Li H., Li Y., Xu Y. (2023). Nitrogen-doped carbon dots from polystyrene for three analytes sensing and their logic recognition. Inorg. Chem. Commun..

[62] Liang L., Wong S.C., Lisak G. (2023). Effects of plastic-derived carbon dots on germination and growth of pea (*Pisum sativum*) via seed nano-priming. Chemosphere.

[63] Abdelhameed M., Elbeh M., Baban N.S., Pereira L., Matula J., Song Y.-A., Ramadi K.B. (2023). High-yield, one-pot upcycling of polyethylene and polypropylene waste into blue-emissive carbon dots. Green Chem..

[64] Kommula B., Banoo M., Roy R.S., Sil S., Sah A.K., Rawat B., Chakraborty S., Meena P., Kailasam K., Gautam U.K. (2023). Landscaping sustainable conversion of waste plastics to carbon dots and enormous diversity in O_2_ harvesting, hypoxia, autophagy. Carbon.

[65] Muro-Hidalgo J.M., Bazany-Rodríguez I.J., Hernández J.G., Pabello V.M.L., Thangarasu P. (2023). Histamine Recognition by Carbon Dots from Plastic Waste and Development of Cellular Imaging: Experimental and Theoretical Studies. J. Fluoresc..

[66] Kumari M., Chaudhary G.R., Chaudhary S., Umar A. (2021). Rapid analysis of trace sulphite ion using fluorescent carbon dots produced from single use plastic cups. Eng. Sci..

[67] Kumari M., Chaudhary S. (2020). Modulating the physicochemical and biological properties of carbon dots synthesised from plastic waste for effective sensing of *E. coli*. Colloids Surf. B Biointerfaces.

[68] Ghosh A., Das G. (2021). Environmentally benign synthesis of fluorescent carbon nanodots using waste PET bottles: Highly selective and sensitive detection of Pb^2+^ ions in aqueous medium. New J. Chem..

[69] Song H., Liu X., Wang B., Tang Z., Lu S. (2019). High production-yield solid-state carbon dots with tunable photoluminescence for white/multi-color light-emitting diodes. Sci. Bull..

[70] Ramanan V., Siddaiah B., Raji K., Ramamurthy P. (2018). Green synthesis of multifunctionalized, nitrogen-doped, highly fluorescent carbon dots from waste expanded polystyrene and its application in the fluorimetric detection of Au^3+^ ions in aqueous media. ACS Sustain. Chem. Eng..

[71] Li S., Hu J., Aryee A.A., Sun Y., Li Z. (2023). Three birds, one stone: Disinfecting and turning waste medical masks into valuable carbon dots for sodium hydrosulfite and Fe^3+^ detection enabled by a simple hydrothermal treatment. Spectrochim. Acta Part A Mol. Biomol. Spectrosc..

[72] Kumari A., Kumar A., Sahu S.K., Kumar S. (2018). Synthesis of green fluorescent carbon quantum dots using waste polyolefins residue for Cu^2+^ ion sensing and live cell imaging. Sens. Actuators B Chem..

[73] Perikala M., Bhardwaj A. (2022). Waste to white light: A sustainable method for converting biohazardous waste to broadband white LEDs. RSC Adv..

[74] Cruz M.I.S.D., Thongsai N., de Luna M.D.G., In I., Paoprasert P. (2019). Preparation of highly photoluminescent carbon dots from polyurethane: Optimization using response surface methodology and selective detection of silver (I) ion. Colloids Surf. A Physicochem. Eng. Asp..

[75] Shaw V., Mondal A., Mondal A., Koley R., Mondal N.K. (2023). Effective utilization of waste plastics towards sustainable control of mosquito. J. Clean. Prod..

[76] Hu Y., Gao Z., Yang J., Chen H., Han L. (2019). Environmentally benign conversion of waste polyethylene terephthalate to fluorescent carbon dots for “on-off-on” sensing of ferric and pyrophosphate ions. J. Colloid Interface Sci..

[77] Wang R., Chen X., Li Q., Zhang A., Ma G., Wei Y., Qu M., Gao L., Wei J. (2023). Solvothermal preparation of nitrogen and phosphorus-doped carbon dots with PET waste as precursor and its application. Mater. Today Commun..

[78] Gu W., Dong Z., Zhang A., Ma T., Hu Q., Wei J., Wang R. (2022). Functionalization of PET with carbon dots as copolymerizable flame retardants for the excellent smoke suppressants and mechanical properties. Polym. Degrad. Stab..

[79] Wei J., Zhang X., Sheng Y., Shen J., Huang P., Guo S., Pan J., Liu B., Feng B. (2014). Simple one-step synthesis of water-soluble fluorescent carbon dots from waste paper. New J. Chem..

[80] Wei J., Shen J., Zhang X., Guo S., Pan J., Hou X., Zhang H., Wang L., Feng B. (2013). Simple one-step synthesis of water-soluble fluorescent carbon dots derived from paper ash. RSC Adv..

[81] Devi S., Gupta R.K., Paul A.K., Tyagi S. (2018). Waste carbon paper derivatized Carbon Quantum Dots/(3-Aminopropyl) triethoxysilane based fluorescent probe for trinitrotoluene detection. Mater. Res. Express.

[82] Park S.J., Park J.Y., Chung J.W., Yang H.K., Moon B.K., Yi S.S. (2020). Color tunable carbon quantum dots from wasted paper by different solvents for anti-counterfeiting and fluorescent flexible film. Chem. Eng. J..

[83] Lin B., Yan Y., Guo M., Cao Y., Yu Y., Zhang T., Huang Y., Wu D. (2018). Modification-free carbon dots as turn-on fluorescence probe for detection of organophosphorus pesticides. Food Chem..

[84] Wang J., Qiu F., Li X., Wu H., Xu J., Niu X., Pan J., Zhang T., Yang D. (2017). A facile one-pot synthesis of fluorescent carbon dots from degrease cotton for the selective determination of chromium ions in water and soil samples. J. Lumin..

[85] Wu Y., Ma G., Zhang A., Gu W., Wei J., Wang R. (2022). Preparation of carbon dots with ultrahigh fluorescence quantum yield based on PET waste. ACS Omega.

[86] Huang P., Xu S., Zhang M., Zhong W., Xiao Z., Luo Y. (2019). Modulation doping of absorbent cotton derived carbon dots for quantum dot-sensitized solar cells. Phys. Chem. Chem. Phys..

[87] Wu Y., Wang R., Xie W., Ma G., Zhang A., Liu B., Huang H., Gao L., Qu M., Wei Y. (2023). Solvent-thermal preparation of sulfur and nitrogen-doped carbon dots with PET waste as precursor and application in light-blocking film. J. Nanoparticle Res..

[88] Vadivel R., Nirmala M., Raji K., Siddaiah B., Ramamurthy P. (2021). Synthesis of highly luminescent carbon dots from postconsumer waste silk cloth and investigation of its electron transfer dynamics with methyl viologen dichloride. J. Indian Chem. Soc..

[89] Chen X., Song Z., Li S., Thang N.T., Gao X., Gong X., Guo M. (2020). Facile one-pot synthesis of self-assembled nitrogen-doped carbon dots/cellulose nanofibril hydrogel with enhanced fluorescence and mechanical properties. Green Chem..

[90] Zhao Z., Guo Y., Zhang T., Ma J., Li H., Zhou J., Wang Z., Sun R. (2020). Preparation of carbon dots from waste cellulose diacetate as a sensor for tetracycline detection and fluorescence ink. Int. J. Biol. Macromol..

[91] Zhao Y., Yang Z., Niu J., Du Z., Federica C., Zhu Z., Yang K., Li Y., Zhao B., Pedersen T.H. (2023). Systematical analysis of sludge treatment and disposal technologies for carbon footprint reduction. J. Environ. Sci..

[92] Kalanidhi K., Nagaraaj P. (2022). A green approach for synthesis of highly fluorescent carbon dots from waste engine oil: A strategy for waste to value added products. Diam. Relat. Mater..

[93] Zhang Y., Wang L., Chen L., Ma B., Zhang Y., Ni W., Tsang D.C. (2021). Treatment of municipal solid waste incineration fly ash: State-of-the-art technologies and future perspectives. J. Hazard. Mater..

[94] Zhang Y., Li H., Gao S., Geng Y., Wu C. (2019). A study on the chemical state of carbon present in fine ash from gasification. Asia-Pac. J. Chem. Eng..

[95] Das T., Saikia B.K., Dekaboruah H., Bordoloi M., Neog D., Bora J.J., Lahkar J., Narzary B., Roy S., Ramaiah D. (2019). Blue-fluorescent and biocompatible carbon dots derived from abundant low-quality coals. J. Photochem. Photobiol. B Biol..

[96] Raj A.M., Chirayil G.T. (2018). Facile synthesis of preformed mixed nano-carbon structure from low rank coal. Manag. Syst. Prod. Eng..

[97] Saikia M., Hower J.C., Das T., Dutta T., Saikia B.K. (2019). Feasibility study of preparation of carbon quantum dots from Pennsylvania anthracite and Kentucky bituminous coals. Fuel.

[98] Jiang Y., Wang Y., Meng F., Wang B., Cheng Y., Zhu C. (2015). N-doped carbon dots synthesized by rapid microwave irradiation as highly fluorescent probes for Pb^2+^ detection. New J. Chem..

[99] Jiang K., Wang Y., Gao X., Cai C., Lin H. (2018). Facile, quick, and gram-scale synthesis of ultralong-lifetime room-temperature-phosphorescent carbon dots by microwave irradiation. Angew. Chem. Int. Ed..

[100] de Medeiros T.V., Manioudakis J., Noun F., Macairan J.-R., Victoria F., Naccache R. (2019). Microwave-assisted synthesis of carbon dots and their applications. J. Mater. Chem. C.

[101] Marinello S., Lolli F., Gamberini R. (2020). Roadway tunnels: A critical review of air pollutant concentrations and vehicular emissions. Transp. Res. Part D Transp. Environ..

[102] Kumar P.G., Lekhana P., Tejaswi M., Chandrakala S. (2021). Effects of vehicular emissions on the urban environment-a state of the art. Mater. Today Proc..

[103] Gu M., Pan Y., Walters W.W., Sun Q., Song L., Wang Y., Xue Y., Fang Y. (2022). Vehicular emissions enhanced ammonia concentrations in winter mornings: Insights from diurnal nitrogen isotopic signatures. Environ. Sci. Technol..

[104] Milku Augustine K., Attiogbe F., Derkyi N., Atepor L. (2023). A Review of Policies and Legislations of Vehicular Exhaust Emissions in Ghana and Their Enforcement. Aerosol Sci. Eng..

[105] Gulia S., Tiwari R., Mendiratta S., Kaur S., Goyal S., Kumar R. (2020). Review of scientific technology-based solutions for vehicular pollution control. Clean Technol. Environ. Policy.

[106] Li Z., Wang L., Li Y., Feng Y., Feng W. (2019). Frontiers in carbon dots: Design, properties and applications. Mater. Chem. Front..

[107] Evode N., Qamar S.A., Bilal M., Barceló D., Iqbal H.M. (2021). Plastic waste and its management strategies for environmental sustainability. Case Stud. Chem. Environ. Eng..

[108] Gu J.-D. (2021). Biodegradability of plastics: The issues, recent advances, and future perspectives. Environ. Sci. Pollut. Res..

[109] Sharuddin S.D.A., Abnisa F., Daud W.M.A.W., Aroua M.K. (2016). A review on pyrolysis of plastic wastes. Energy Convers. Manag..

[110] Maqsood T., Dai J., Zhang Y., Guang M., Li B. (2021). Pyrolysis of plastic species: A review of resources and products. J. Anal. Appl. Pyrolysis.

[111] Vassilev S.V., Kitano K., Takeda S., Tsurue T. (1995). Influence of mineral and chemical composition of coal ashes on their fusibility. Fuel Process. Technol..

[112] Naik V.M., Bhosale S.V., Kolekar G.B. (2022). A brief review on the synthesis, characterisation and analytical applications of nitrogen doped carbon dots. Anal. Methods.

[113] Dhenadhayalan N., Lin K.C., Saleh T.A. (2020). Recent advances in functionalized carbon dots toward the design of efficient materials for sensing and catalysis applications. Small.

[114] National Clothing Product Stewardship Schemes. https://ausfashioncouncil.com/wp-content/uploads/2023/05/AFC-NCPSS-Data-Report.pdf.

[115] Wang C., Xia K., Zhang Y., Kaplan D.L. (2019). Silk-based advanced materials for soft electronics. Acc. Chem. Res..

[116] He H., Zhang Y., Wang P., Hu D. (2021). Preparation of sponge-cake-like N-doped porous carbon materials derived from silk fibroin by chemical activation. Microporous Mesoporous Mater..

[117] Munusamy S., Mandlimath T.R., Swetha P., Al-Sehemi A.G., Pannipara M., Koppala S., Shanmugam P., Boonyuen S., Pothu R., Boddula R. (2023). Nitrogen-doped carbon dots: Recent developments in its fluorescent sensor applications. Environ. Res..

[118] Li W., Wang S., Li Y., Ma C., Huang Z., Wang C., Li J., Chen Z., Liu S. (2017). One-step hydrothermal synthesis of fluorescent nanocrystalline cellulose/carbon dot hydrogels. Carbohydr. Polym..

[119] Shen P., Gao J., Cong J., Liu Z., Li C., Yao J. (2016). Synthesis of cellulose-based carbon dots for bioimaging. ChemistrySelect.

[120] Liao X., Chen C., Zhou R., Huang Q., Liang Q., Huang Z., Zhang Y., Hu H., Liang Y. (2020). Comparison of N-doped carbon dots synthesized from the main components of plants including cellulose, lignin, and xylose: Characterized, fluorescence mechanism, and potential applications. Dye Pigment..

[121] Huang H., Ge H., Ren Z., Huang Z., Xu M., Wang X. (2021). Controllable synthesis of biocompatible fluorescent carbon dots from cellulose hydrogel for the specific detection of Hg^2+^. Front. Bioeng. Biotechnol..

[122] Wu P., Li W., Wu Q., Liu Y., Liu S. (2017). Hydrothermal synthesis of nitrogen-doped carbon quantum dots from microcrystalline cellulose for the detection of Fe^3+^ ions in an acidic environment. RSC Adv..

[123] Zhang W., Li L., Yan M., Ma J., Wang J., Liu C., Bao Y., Jin H., Fan Q. (2023). Turning waste into treasure: Multicolor carbon dots synthesized from waste leather scrap and their application in anti-counterfeiting. ACS Sustain. Chem. Eng..

[124] Craciun A., Diac A., Focsan M., Socaci C., Magyari K., Maniu D., Mihalache I., Veca L., Astilean S., Terec A. (2016). Surface passivation of carbon nanoparticles with p-phenylenediamine towards photoluminescent carbon dots. RSC Adv..

[125] Kaur M., Kaur M., Sharma V.K. (2018). Nitrogen-doped graphene and graphene quantum dots: A review onsynthesis and applications in energy, sensors and environment. Adv. Colloid Interface Sci..

[126] Xu Y., Wang C., Sui L., Ran G., Song Q. (2023). Phosphoric acid densified red emissive carbon dots with a well-defined structure and narrow band fluorescence for intracellular reactive oxygen species detection and scavenging. J. Mater. Chem. C.

[127] Goswami J., Rohman S.S., Guha A.K., Basyach P., Sonowal K., Borah S.P., Saikia L., Hazarika P. (2022). Phosphoric acid assisted synthesis of fluorescent carbon dots from waste biomass for detection of Cr (VI) in aqueous media. Mater. Chem. Phys..

[128] Zhang Y., Tan B., Zhang X., Guo L., Zhang S. (2021). Synthesized carbon dots with high N and S content as excellent corrosion inhibitors for copper in sulfuric acid solution. J. Mol. Liq..

[129] Zhang Y., Zhang S., Tan B., Guo L., Li H. (2021). Solvothermal synthesis of functionalized carbon dots from amino acid as an eco-friendly corrosion inhibitor for copper in sulfuric acid solution. J. Colloid Interface Sci..

[130] Liang Y., Xu D., Feng P., Hao B., Guo Y., Wang S. (2021). Municipal sewage sludge incineration and its air pollution control. J. Clean. Prod..

[131] Rangabhashiyam S., dos Santos Lins P.V., de Magalhães Oliveira L.M., Sepulveda P., Ighalo J.O., Rajapaksha A.U., Meili L. (2022). Sewage sludge-derived biochar for the adsorptive removal of wastewater pollutants: A critical review. Environ. Pollut..

[132] Chen X., Jeyaseelan S., Graham N. (2002). Physical and chemical properties study of the activated carbon made from sewage sludge. Waste Manag..

[133] Ding A., Zhang R., Ngo H.H., He X., Ma J., Nan J., Li G. (2021). Life cycle assessment of sewage sludge treatment and disposal based on nutrient and energy recovery: A review. Sci. Total Environ..

[134] Zu F., Yan F., Bai Z., Xu J., Wang Y., Huang Y., Zhou X. (2017). The quenching of the fluorescence of carbon dots: A review on mechanisms and applications. Microchim. Acta.

[135] Bhat S.A., Hassan T., Majid S. (2019). Heavy metal toxicity and their harmful effects on living organisms–a review. Int. J. Med. Sci. Diagn. Res..

[136] Wu Y., Chen X., Wu W. (2023). Multiple Stimuli-Response Polychromatic Carbon Dots for Advanced Information Encryption and Safety. Small.

[137] Ji C., Xu W., Han Q., Zhao T., Deng J., Peng Z. (2023). Light of Carbon: Recent Advancements of Carbon Dots for LEDs. Nano Energy.

[138] Olabi A., Abdelkareem M.A. (2022). Renewable energy and climate change. Renew. Sustain. Energy Rev..

[139] Hu J., Jia W., Wu X., Zhang H., Wang Y., Liu J., Yang Y., Tao S., Wang X. (2022). Carbon dots can strongly promote photosynthesis in lettuce (*Lactuca sativa* L.). Environ. Sci. Nano.

[140] Guo B., Liu G., Wei H., Qiu J., Zhuang J., Zhang X., Zheng M., Li W., Zhang H., Hu C. (2022). The role of fluorescent carbon dots in crops: Mechanism and applications. SmartMat.

